# Tobacco Product Use Among Military Veterans — United States, 2010–2015

**DOI:** 10.15585/mmwr.mm6701a2

**Published:** 2018-01-12

**Authors:** Satomi Odani, Israel T. Agaku, Corinne M. Graffunder, Michael A. Tynan, Brian S. Armour

**Affiliations:** 1 Office on Smoking and Health, National Center for Chronic Disease Prevention and Health Promotion, CDC.

In 2015, an estimated 18.8 million U.S. adults were military veterans (*1*). Although the prevalence of tobacco-attributable conditions is high among veterans (*2*), there is a paucity of data on use of tobacco products, other than cigarettes, in this population. To monitor tobacco product use among veterans, CDC analyzed self-reported current (i.e., past 30-day) use of five tobacco product types (cigarettes, cigars [big cigars, cigarillos, or little cigars], roll-your-own tobacco, pipes, and smokeless tobacco [chewing tobacco, snuff, dip, or snus]) from the National Survey on Drug Use and Health (NSDUH). Overall, 29.2% of veterans reported current use of any of the assessed tobacco products. Cigarettes were the most commonly used tobacco product (21.6%), followed by cigars (6.2%), smokeless tobacco (5.2%), roll-your-own tobacco (3.0%), and pipes (1.5%); 7.0% of veterans currently used two or more tobacco products. Within subgroups of veterans, current use of any of the assessed tobacco products was higher among persons aged 18–25 years (56.8%), Hispanics (34.0%), persons with less than a high school diploma (37.9%), those with annual family income <$20,000 (44.3%), living in poverty (53.7%), reporting serious psychological distress (48.2%), and with no health insurance (60.1%). By age and sex subgroups, use of any of the assessed tobacco products was significantly higher among all veteran groups than their nonveteran counterparts, except males aged ≥50 years. Expanding the reach of evidence-based tobacco control interventions among veterans could reduce tobacco use prevalence in this population.

NSDUH is an annual, in-person survey of the civilian, noninstitutionalized U.S. population aged ≥12 years conducted at the respondent’s residence (*3*). The analyses in this report were restricted to adults aged ≥18 years. Data were pooled for 2010–2015 to increase statistical precision of estimates. Pooled sample size for adults aged ≥18 years was 238,917; annual response rate averaged 65.4%.*

Military veterans were those who had “ever been in the United States Armed Forces” and were “now separated/retired from reserves/active duty” (pooled n = 13,140). Nonveterans were those who had never been in the United States Armed Forces (pooled n = 224,648).^†^ Respondents who reported currently being in a reserve component, or did not provide an answer were excluded from the analyses. Current users of cigarettes, cigars, roll-your-own tobacco, pipes, and smokeless tobacco were persons who had used the respective products during the past 30 days. Any tobacco product use was defined as use of any of the five assessed tobacco product types. Respondents who reported use of two or more tobacco product types during the past 30 days were further classified as current users of two or more tobacco product types.^§^ Prevalence estimates were calculated overall and by sex, age, race/ethnicity, education, annual family income, poverty status,^¶^ marital status, presence of serious psychological distress,** and health insurance coverage.^††^ Additionally, age- and sex-specific prevalence estimates were calculated among veterans and nonveterans separately to allow direct comparisons of the two groups, given differences between veterans and nonveterans by age and sex.^§§^ Cigarette quit ratio was calculated as the proportion of former cigarette smokers (persons who smoked ≥100 cigarettes during lifetime, but did not smoke in past 12 months) among ever cigarette smokers (persons who smoked ≥100 cigarettes during lifetime); quit ratios were not calculated for the other noncigarette tobacco products because of the absence of lifetime usage thresholds to distinguish actual former users from experimenters. The proportion of former cigarette smokers who still reported current (past 30-day) use of any noncigarette tobacco product (cigars, roll-your-own tobacco, pipes, and smokeless tobacco) was computed to determine complete tobacco abstinence among those who had quit cigarette smoking. Within-group differences and differences between veterans and nonveterans were assessed with Chi-squared tests, and trends in estimates were tested with logistic regression using orthogonal polynomials, with statistical significance at p<0.05. Estimates with relative standard errors ≥30% were suppressed.

Among veterans overall, 29.2% reported current use of any tobacco product, and 7.0% reported current use of two or more tobacco products (Table 1). By tobacco product type, current use was highest for cigarettes (21.6%), followed by cigars (6.2%), smokeless tobacco (5.2%), roll-your-own tobacco (3.0%), and pipes (1.5%). Significant differences existed within veteran subgroups in current use of any tobacco product. Prevalence was lowest among persons who were aged ≥50 years (23.8%), non-Hispanic white (28.3%), had a college degree or higher (17.2%), an annual family income of ≥$75,000 (23.9%), living at more than twice the Federal Poverty Threshold (25.2%), married (24.3%), did not report serious psychological distress (28.5%), and were insured (27.3%). Prevalence was highest among persons who were aged 18–25 years (56.8%), Hispanic (34.0%), had less than a high school diploma (37.9%), an annual family income of <$20,000 (44.3%), were living in poverty (53.7%), were never married (43.4%), who reported serious psychological distress (48.2%), and who were uninsured (60.1%).

**TABLE 1:** Point prevalence estimates and 95% confidence intervals of past 30-day use of tobacco product among military veterans* aged ≥18 years, overall and by sociodemographic characteristics — National Survey on Drug Use and Health, United States, 2010–2015

Characteristic	Cigarettes % (95% CI)	Cigars (big cigars/cigarillos/little cigars) % (95% CI)	Roll-your-own tobacco % (95% CI)	Pipe % (95% CI)	Smokeless tobacco (chewing tobacco/snuff/dip/snus) % (95% CI)	Any tobacco product^¶^ % (95% CI)	≥2 tobacco products** % (95% CI)
**Overall (n = 13,140)**	21.6 (20.7–22.6)	6.2 (5.7–6.8)	3.0 (2.7–3.4)	1.5 (1.2–1.7)	5.2 (4.7–5.7)	29.2 (28.1–30.2)	7.0 (6.4–7.5)
**Sex**							
Male	21.1 (20.1–22.1)^†^	6.5 (5.9–7.1)^†^	3.0 (2.6–3.4)	1.6 (1.3–1.9)^†^	5.6 (5.1–6.1)^†^	29.1 (28.0–30.2)	7.1 (6.5–7.7)^†^
Female	28.9 (25.3–32.5)^†^	2.1 (1.3–2.9)^†^	3.4 (1.9–5.0)	—^§^	—^§^	29.7 (26.1–33.3)	4.8 (3.1–6.5)^†^
**Age group (yrs)**							
18–25	47.3 (43.5–51.2)^†^	13.3 (10.7–16.0)^†^	5.3 (3.8–6.7)^†^	2.5 (1.2–3.8)	15.4 (12.7–18)^†^	56.8 (52.9–60.6)^†^	21.2 (18.1–24.3)^†^
26–34	43.7 (40.2–47.2)^†^	11.2 (9.0–13.4)^†^	6.0 (4.5–7.4)^†^	1.6 (0.7–2.4)	12 (9.8–14.2)^†^	52.7 (49.1–56.2)^†^	17.6 (15–20.2)^†^
35–49	31.5 (29.4–33.6)^†^	8.8 (7.4–10.1)^†^	3.8 (3.0–4.6)^†^	1.1 (0.6–1.5)	11.3 (9.8–12.7)^†^	43.2 (41.0–45.5)^†^	10.8 (9.4–12.3)^†^
≥50	17.3 (16.2–18.5)^†^	5.2 (4.5–5.8)^†^	2.6 (2.2–3.0)^†^	1.5 (1.2–1.9)	3.2 (2.7–3.7)^†^	23.8 (22.5–25.1)^†^	5.0 (4.4–5.7)^†^
**Race/Ethnicity**							
Non-Hispanic white	20.2 (19.2–21.2)^†^	5.9 (5.3–6.5)^†^	2.9 (2.5–3.3)	1.5 (1.2–1.9)	5.8 (5.2–6.3)^†^	28.3 (27.1–29.4)^†^	6.7 (6.0–7.3)
Non-Hispanic black	26.3 (23.2–29.4)^†^	9.4 (7.4–11.4)^†^	3.6 (2.2–4.9)	1.2 (0.5–1.9)	1.9 (1.1–2.8)^†^	32.1 (28.7–35.4)^†^	8.3 (6.4–10.1)
Hispanic	29.1 (24.1–34.1)^†^	6.0 (3.8–8.3)^†^	—^§^	—^§^	4.7 (2.8–6.6)^†^	34.0 (28.9–39.1)^†^	7.7 (5.0–10.3)
Non-Hispanic other	29.0 (22.8–35.2)^†^	—^§^	5.4 (2.9–7.9)	—^§^	3.2 (1.8–4.5)^†^	33.6 (27.1–40.0)^†^	8.6 (5.7–11.4)
**Education**							
Less than high school	30.4 (26.6–34.1)	6.6 (4.6–8.7)^†^	6.1 (4.2–8.0)^†^	2.8 (1.5–4.1)	6.3 (4.4–8.2)^†^	37.9 (34.0–41.9)^†^	10.4 (8–12.7)^†^
High school	26.3 (24.5–28.1)	5.9 (4.9–6.9)^†^	4.2 (3.4–4.9)^†^	1.4 (0.9–1.9)	6.3 (5.4–7.2)^†^	33.9 (31.9–35.8)^†^	8.8 (7.7–9.9)^†^
Some college	25.7 (23.8–27.5)	6.9 (5.9–7.9)^†^	3.3 (2.6–4.0)^†^	1.4 (0.9–1.8)	6.1 (5.2–6.9)^†^	33.6 (31.6–35.5)^†^	7.9 (6.9–9.0)^†^
College degree or higher	10.1 (8.7–11.5)	5.8 (4.7–6.8)^†^	0.7 (0.4–1.1)^†^	1.3 (0.8–1.8)	2.9 (2.1–3.6)^†^	17.2 (15.5–18.9)^†^	3.0 (2.2–3.8)^†^
**Annual family income ($)**							
<$20,000	37.7 (34.5–40.9)^†^	8.2 (6.6–9.9)^†^	10.3 (8.4–12.3)^†^	3.0 (1.9–4.0)^†^	5.2 (3.9–6.6)	44.3 (41.0–47.6)^†^	15.9 (13.6–18.1)^†^
$20,000–$49,999	24.8 (23.0–26.5)^†^	5.6 (4.7–6.5)^†^	3.5 (2.8–4.2)^†^	1.6 (1.1–2.1)^†^	4.9 (4.1–4.9)	31.5 (29.6–33.3)^†^	7.5 (6.5–7.5)^†^
$50,000–$74,999	18.7 (16.7–20.8)^†^	5.6 (4.3–6.8)^†^	1.5 (0.8–2.1)^†^	1.6 (0.9–2.3)^†^	4.6 (3.7–4.6)	25.8 (23.5–28.1)^†^	4.9 (3.8–4.9)^†^
>$75,000	15.0 (13.5–16.4)^†^	6.6 (5.6–7.6)^†^	1.1 (0.7–1.4)^†^	0.8 (0.5–1.1)^†^	5.8 (4.9–6.7)	23.9 (22.1–25.6)^†^	4.6 (3.8–5.5)^†^
**Poverty status^††^**							
Living in poverty	46.2 (41.9–50.5)^†^	9.9 (7.5–12.3)^†^	14.1 (11.1–17.2)^†^	3.2 (1.8–4.6)^†^	7.4 (5.2–9.6)^†^	53.7 (49.4–58.1)^†^	21.0 (17.5–24.4)^†^
Up to 2X Federal Poverty Threshold	32.0 (29.3–34.6)^†^	6.5 (5.2–7.9)^†^	5.6 (4.4–6.8)^†^	1.8 (1.0–2.6)^†^	5.7 (4.6–6.8)^†^	38.7 (35.9–41.4)^†^	10.6 (9.0–12.3)^†^
More than 2X Federal Poverty Threshold	17.5 (16.5–18.6)^†^	5.9 (5.2–6.5)^†^	1.6 (1.3–1.9)^†^	1.3 (1.0–1.6)^†^	5.0 (4.4–5.5)^†^	25.2 (24.1–26.4)^†^	5.1 (4.5–5.6)^†^
**Marital status**							
Married	16.6 (15.5–17.7)^†^	5.6 (4.9–6.3)^†^	2.1 (1.7–2.5)^†^	1.1 (0.8–1.3)^†^	5.1 (4.5–5.7)^†^	24.3 (23.1–25.6)^†^	5.2 (4.6–5.9)^†^
Widowed/Divorced/Separated	30.4 (28.2–32.6)^†^	6.7 (5.5–7.9)^†^	4.9 (4.0–5.9)^†^	2.6 (1.8–3.4)^†^	4.8 (4.0–5.7)^†^	37.4 (35.1–39.8)^†^	9.6 (8.2–10.9)^†^
Never married	36.1 (33.0–39.3)^†^	9.9 (8.0–11.8)^†^	5.2 (4.1–6.3)^†^	1.5 (0.8–2.1)^†^	7.4 (5.8–8.9)^†^	43.4 (40.1–46.8)^†^	12.9 (11.0–14.8)^†^
**Serious psychological distress** ^§§^							
No	21.0 (20.0–22.0)^†^	6.1 (5.6–6.7)^†^	2.8 (2.5–3.2)^†^	1.4 (1.1–1.7)^†^	5.2 (4.7–5.7)	28.5 (27.4–29.6)^†^	6.7 (6.1–7.2)^†^
Yes	40.8 (35.0–46.5)^†^	9.4 (6.1–12.7)^†^	9.2 (6.2–12.2)^†^	4.1 (1.9–6.3)^†^	6.6 (4.4–8.8)	48.2 (42.2–54.2)^†^	15.7 (11.9–19.5)^†^
**Health insurance coverage** ^¶¶^							
Uninsured	51.4 (46.7–56.1)^†^	12.0 (9.4–14.5)^†^	8.8 (6.7–10.8)^†^	2.6 (1.3–4.0)^†^	10.5 (7.8–13.2)^†^	60.1 (55.4–64.8)^†^	19.4 (16.2–22.6)^†^
Insured	19.8 (18.9–20.8)^†^	5.9 (5.3–6.5)^†^	2.7 (2.3–3.1)^†^	1.4 (1.1–1.7)^†^	4.9 (4.4–5.4)^†^	27.3 (26.2–28.4)^†^	6.2 (5.6–6.8)^†^

**Abbreviation**: CI = confidence interval.

*Persons who reported having ever been in the U.S. Armed Forces and currently being separated or retired from reserves/active duty at the time of the survey (pooled n = 13,140).

^†^ Estimates significantly varied within sociodemographic subgroups (p<0.05).

^§^ Estimates not presented because of relative standard error ≥30%.

^¶^ Any tobacco product–users were persons who reported past-30 day use of at least one of the five tobacco product types (cigarettes, cigars, roll-your-own tobacco, pipe, and smokeless tobacco). Respondents who had at least one missing response to any of the tobacco product use questions were excluded from the analysis (76, 0.03% of respondents).

** ≥2 tobacco product–users were persons who reported past-30 day use of ≥2 tobacco products. Respondents who had at least one missing response to any of the tobacco product use questions were excluded from the analysis (76, 0.03% of respondents).

^††^ Poverty status indicates a person’s family income relative to Federal Poverty Threshold. https://www.census.gov/data/tables/time-series/demo/income-poverty/historical-poverty-thresholds.html.

^§§^ The Kessler Serious Psychological Distress is a series of six questions that asks about feelings of sadness, nervousness, restlessness, worthlessness, hopelessness, and feeling like everything is an effort during the past 30 days. Participants responded using a Likert Scale ranging from “None of the time” (score = 0) to “All of the time” (score = 4). Responses were summed over the six questions for a total possible score of 0–24; respondents with a score ≥13 were coded as having serious psychological distress, and respondents with a score <13 were coded as not having serious psychological distress. https://jamanetwork.com/journals/jamapsychiatry/fullarticle/207204.

^¶¶^ Respondents were classified as being insured if they had private insurance, Medicare, Medicaid/HIPCOV, Champus, ChampVA, VA, Military, or other health insurance. Among veterans, weighted proportions of those insured and uninsured were 94.7% and 5.7%, respectively.

The prevalence of current use of any tobacco product was significantly higher among veterans than nonveterans in all age and sex strata, except males aged ≥50 years (Table 2). Among both veterans and nonveterans, the prevalence of any tobacco product use was significantly higher among males than among females in each age stratum, except veterans aged ≥50 years.

**TABLE 2:** Comparisons of age and sex-specific point prevalence estimates of past 30-day use of tobacco product between military veterans* and nonveterans — National Survey on Drug Use and Health, United States, 2010–2015

Age group, yrs (sex)	Cigarettes % (95% CI)	Cigars (big cigars/cigarillos/little cigars) % (95% CI)	Roll-your-own tobacco % (95% CI)	Pipe % (95% CI)	Smokeless tobacco (chewing tobacco/snuff/dip/snus) % (95% CI)	Any tobacco product^¶^ % (95% CI)	≥2 tobacco products** % (95% CI)
**Veterans (n = 13,140)**							
18–25 (male)	50.2 (45.8–54.5)**^†^**	14.7 (11.6–17.8)	5.6 (3.9–7.4)	3.2 (1.5–4.8)	18.9 (15.7–22.2)**^†^**	61.7 (57.4–66.0)**^†^**	23.7 (20.1–27.4)**^†^**
18–25 (female)	36.4 (28.8–44.0)**^†^**	8.0 (3.4–12.5)	—^§^	—^§^	—^§^	37.9 (30.2–45.5)**^†^**	11.4 (6.4–16.4)**^†^**
26–34 (male)	45.5 (41.6–49.5)**^†^**	12.7 (10–15.3)	6.2 (4.6–7.9)	1.8 (0.8–2.8)	14.0 (11.4–16.6)**^†^**	55.9 (51.9–59.8)**^†^**	19.3 (16.3–22.4)**^†^**
26–34 (female)	35.2 (28.2–42.3)**^†^**	—^§^	—^§^	—^§^	—^§^	37.4 (30.3–44.5)**^†^**	9.5 (5.3–13.7)**^†^**
35–49 (male)	31.5 (29.2–33.7)**^†^**	9.6 (8.2–11.1)**^†^**	4.0 (3.1–4.8)	1.2 (0.7–1.8)	12.9 (11.3–14.5)**^†^**	44.8 (42.3–47.2)**^†^**	11.9 (10.3–13.5)**^†^**
35–49 (female)	31.5 (26.3–36.7)**^†^**	—^§^	—^§^	—^§^	—	32.7 (27.5–38.0)**^†^**	3.5 (1.7–5.3)
≥50 (male)	17.0 (15.8–18.1)	5.4 (4.7–6.1)	2.6 (2.1–3.0)**^†^**	1.6 (1.2–2.0)	3.3 (2.8–3.9)	23.7 (22.5–25.0)	5.1 (4.4–5.7)
≥50 (female)	24.8 (18.8–30.8)**^†^**	—^§^	—^§^	—^§^	—^§^	24.9 (10.9–30.9)**^†^**	—^§^
**Nonveterans (n = 224,648)**							
18–25 (male)	35.3 (34.7–35.9)**^†^**	15.2 (14.7–15.6)	6.7 (6.4–7.0)	2.7 (2.5–2.9)	10.4 (10.1–10.8)**^†^**	45.3 (44.7–45.9)**^†^**	18.8 (18.3–19.3)**^†^**
18–25 (female)	26.0 (25.5–26.5)**^†^**	5.4 (5.1–5.6)	3.5 (3.3–3.7)	1.1 (1.0–1.2)	0.7 (0.6–0.7)	28.8 (28.3–29.3)**^†^**	6.5 (6.3–6.8)**^†^**
26–34 (male)	36.3 (35.3–37.3)**^†^**	11.5 (10.8–12.2)	5.9 (5.5–6.4)	1.4 (1.2–1.7)	8.4 (7.9–9.0)**^†^**	45.2 (44.2–46.3)**^†^**	14.8 (14.1–15.5)**^†^**
26–34 (female)	26.7 (25.9–27.5)**^†^**	3.1 (2.8–3.4)	3.0 (2.7–3.2)	0.4 (0.3–0.5)	0.5 (0.3–0.6)	28.3 (27.5–29.1)**^†^**	4.6 (4.3–5.0)**^†^**
35–49 (male)	26.3 (25.5–27.1)**^†^**	7.3 (6.9–7.8)**^†^**	4.5 (4.2–4.8)	0.9 (0.8–1.1)	7.8 (7.3–8.2)**^†^**	35.6 (34.7–36.4)**^†^**	9.3 (8.8–9.8)**^†^**
35–49 (female)	23.0 (22.3–23.6)**^†^**	1.8 (1.6–2.0)	2.9 (2.7–3.2)	0.2 (0.1–0.2)	0.3 (0.2–0.4)	23.8 (23.2–24.4)**^†^**	3.9 (3.6–4.2)
≥50 (male)	18.1 (17.2–18.9)	5.7 (5.2–6.2)	3.3 (2.9–3.7)	1.3 (1.1–1.6)	3.7 (3.3–4.1)	25.1 (24.2–26.1)	5.7 (5.2–6.2)
≥50 (female)	14.8 (14.2–15.3)**^†^**	0.6 (0.5–0.7)	2.0 (1.8–2.2)	0.1 (0.1–0.2)	0.4 (0.3–0.6)	15.4 (14.8–16.0)**^†^**	2.4 (2.2–2.6)

**Abbreviation**: CI = confidence interval.

* Veterans were persons who reported having ever been in the U.S. Armed Forces and being separated or retired from reserves/active duty at the time of the survey (pooled n = 13,140). Nonveterans were persons who reported having never been in the U.S. Armed Forces (pooled n = 224,648).

^†^ Estimates significantly different from corresponding estimate among veteran and nonveteran populations.

^§^ Estimates not presented because of relative standard error ≥30%.

^¶^ Any tobacco product users were persons who reported past-30 day use of at least one of the five tobacco product types (cigarette, cigar, roll-your-own tobacco, pipe, and smokeless tobacco). Respondents who had at least one missing response to any of the tobacco product use question were excluded from the analysis (76, 0.03% of respondents).

** ≥2 tobacco-product–users were persons who reported past-30 day use of ≥2 tobacco products. Respondents who had at least one missing response to any of the tobacco product use questions were excluded from the analysis (76, 0.03% of respondents).

Cigarette quit ratio estimates were not significantly different among veterans and nonveterans in any age/sex stratum except females aged 18–25 years (18.7%, veterans versus 10.4% nonveterans), females aged ≥50 years (50.8% versus 62.1%); and males aged ≥50 years (72.4% versus 61.1%,) (p<0.05) (Figure). For both veterans and nonveterans, sex-specific quit ratios increased with increasing age (p<0.05 for trend). Current use of noncigarette tobacco products among former cigarette smokers was not significantly different among veterans and nonveterans in any age/sex stratum except males aged 35–49 years (26.4% versus 17.9%, veterans versus nonveterans), and males aged ≥50 years (8.6% versus 11.7%) (p<0.05). Although sex-specific prevalence of noncigarette tobacco product use decreased with increasing age among nonveterans (p<0.05 for trend), trends were not significant for veterans.

**FIGURE:**
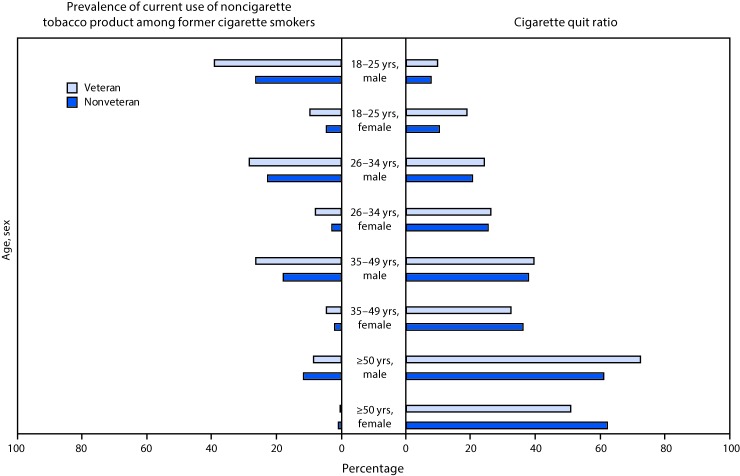
Prevalence of current (past 30-day) use of noncigarette tobacco product* among former cigarette smokers and cigarette quit ratios,^†^ among military veterans and nonveterans,^§^ by age and sex — National Survey on Drug Use and Health, United States, 2010–2015 * Noncigarette tobacco product includes cigars, roll-your-own tobacco, pipes, and smokeless tobacco. ^†^ Cigarette quit ratio was calculated as the proportion of former smokers (persons who smoked ≥100 cigarettes during lifetime and did not smoke in the past 12 months) among ever smokers (persons who smoked ≥100 cigarettes during lifetime). ^§^ Veterans were individuals who reported having ever been in the U.S. Armed Forces and currently being separated or retired from reserves/active duty at the time of the survey (pooled n = 13,140). Nonveterans were individuals who reported having never been in the U.S. Armed Forces (pooled n = 224,648). Prevalence of current use of noncigarette tobacco product among former smokers was significantly different among veterans and nonveterans in males aged 35–49 years and males aged ≥50 years (p<0.05). Cigarette quit ratios were significantly different among veterans and nonveterans in females aged 18–25 years; males aged ≥50 years; and females aged ≥50 years (p<0.05).

## Discussion

During 2010–2015, close to three in 10 U.S. veterans were current users of any tobacco products, and prevalence of use of any tobacco product was higher among veterans than among nonveterans within all subgroups of age and sex, except males aged ≥50 years. Evidence-based strategies can help veterans quit tobacco use, including quitline services (e.g., 1–855-QUIT-VET and 1–800-QUIT-NOW^¶¶^); text messaging services (e.g., https://www.publichealth.va.gov/smoking/smokefreevet.asp); web resources (e.g., https://www.publichealth.va.gov/smoking/ and https://smokefree.gov/veterans); group/individual counseling; and use of FDA approved cessation medications. Additionally, CDC’s Tips From Former Smokers’ Campaign (https://www.cdc.gov/tobacco/campaign/tips/index.html) features real stories of smokers, including military service members and veterans who live with smoking-related diseases and disabilities, to motivate smokers to quit.***

Despite similar quit ratios among veterans and nonveterans, the prevalence of current cigarette smoking was higher among veterans in most age groups. These findings are consistent with those of previous studies showing high rates of smoking initiation among military personnel (*4*,*5*). Approximately 38% of current military smokers initiate tobacco use after enlisting in military service (*6*). Factors encouraging or enabling tobacco use in the military include stress, peer influence, and easy access to cheap tobacco products (*7*,*8*).

The high prevalence of tobacco use among military personnel and veterans also has a significant financial impact. During 2010, the Veterans Health Administration (VHA) spent an estimated $2.7 billion (7.6% of the VHA expenditures on health services for which the cost of smoking could be attributed) on smoking-related ambulatory care, prescription drugs, hospitalization, and home health care for the segment of the veteran population receiving VHA services (*2*). Tobacco use among active military personnel can eventually contribute to VHA expenditures as these become veterans. Reducing tobacco use among both active duty military and veterans can therefore result in a substantial reduction in tobacco-related morbidity and mortality and billions of dollars in savings from averted medical costs.

Implementation of evidence-based tobacco control interventions among military and veteran populations can help reduce prevalence by preventing initiation and relapse, and encouraging quitting. Because more than a third of current smokers in active duty military initiate smoking after enlistment (*6*), and because veterans continue to have access to military installations after retirement from the military, interventions that impact both current and former military members are important to reduce tobacco use among veterans. Strategies could include promoting cessation to current military personnel and veterans, implementing tobacco-free policies at military installations and Veterans Affairs medical centers and clinics, increasing the age requirement to buy tobacco on military bases to 21 years, and eliminating tobacco product discounts through military retailers (*9*,*10*).

The findings in this report are subject to at least five limitations. First, these cross-sectional data do not allow a comparison of prevalence estimates for the same cohort as they age. Second, the definition of veterans used in this study possibly includes persons who served in the U.S. Armed Forces but might not meet the statutory definition of “veterans” (e.g., dishonorably discharged persons). Third, data were not available for newer tobacco products such as hookah and electronic cigarettes. Fourth, these analyses used data pooled from multiple years; therefore, only time-averaged prevalence estimates could be assessed. Finally, multivariable analyses were not performed to identify independent predictors of tobacco use, especially among subgroups where multiple risk factors for tobacco use might exist simultaneously.

The health and economic costs of tobacco use among veterans are high (*2*). Opportunities exist to make tobacco products less acceptable and accessible for both active duty military personnel and veterans. For example, U.S. Department of Veterans Affairs health care facilities are required by Federal law to have designated smoking areas.^†††^ Progress has been made in recent years in promoting tobacco cessation and denormalizing smoking among military personnel and veterans. This includes VHA’s efforts to increase access to tobacco use treatment options^§§§^ as well as the U.S. Department of Defense’s (DOD) prohibition of tobacco use on DOD medical campuses and medical treatment facilities, with a goal to achieve tobacco-free installations by 2020.^¶¶¶^ Continued implementation of these and other evidence-based tobacco control interventions on military and veteran facilities can help reduce tobacco use and tobacco-attributable disease and death among veterans.

**Summary**

**What is already known about this topic?**

In the United States, the prevalence of adverse health conditions caused by tobacco use is particularly high among veterans; however, data on use of tobacco products other than cigarettes in this population are limited.

**What is added by this report?**

Analysis of data from the 2010–2015 National Survey on Drug Use and Health indicates that 29.2% of veterans reported current tobacco product use. Cigarettes were the most commonly used tobacco product (21.6%), followed by cigars (6.2%), smokeless tobacco (5.2%), roll-your-own tobacco (3.0%), and pipes (1.5%); 7.0% of veterans currently used two or more tobacco products. Within veteran subgroups, current use of any of the assessed tobacco products was higher among persons aged 18–25 years (56.8%), Hispanics (34.0%), persons who had not completed high school (37.9%), whose annual family income was <$20,000 (44.3%), were living in poverty (53.7%), who reported serious psychological distress (48.2%), and who had no health insurance (60.1%). By age and sex subgroups, any tobacco product use was significantly higher among all veteran groups than their nonveteran counterparts, except males aged ≥50 years.

**What are the implications for public health practice?**

Evidence-based tobacco control interventions can be implemented to reach veterans, which could reduce tobacco use prevalence and tobacco-attributable disease and death among this population. Strategies could include promoting cessation to current military personnel and veterans, implementing tobacco-free policies at military installations and Veterans Affairs medical centers and clinics, increasing the age requirement to buy tobacco on military bases to 21 years, and eliminating tobacco product discounts through military retailers.

## Footnotes

* Data are collected annually through handheld computer-assisted face-to-face interviews, using a combination of interviewer-administered and respondent self-administered questions. Sample sizes and response rates for adult population were 39,069; 65.9% (2010); 38,965; 64.7% (2011); 37,657; 67.1% (2012); 37,250; 64.5% (2013); 41,520; 61.2% (2014), and 43,401; 69.2% (2015). Of the 238,917 adults in the pooled sample, 13,140 were veterans, 224,648 were nonveterans, 1,040 were currently in a reserve component, and 89 did not provide an answer.

† To determine military veteran status, respondents were asked two questions. The first question was “Have you ever been in the United States Armed Forces?” Categorical response options were “Yes” or “No.” Those who answered “Yes” were then asked “Are you currently on active duty in the United States Armed Forces, are you in a Reserve component, or are you now separated or retired from the military?” Categorical response options were “On active duty in the Armed Forces,” “In a reserve component” or “Now separated/retired from reserves/active duty.” Persons who reported currently being on active duty were not included in the survey. Respondents who reported currently being in a reserve component (1,040; 0.4% of respondents) and those did not provide an answer (89; 0.04% of respondents) were excluded from the analysis.

§ For the use of any tobacco product types and two or more tobacco product types, respondents who had at least one missing response to any of the five tobacco product type questions were excluded from the analysis (76; 0.03% of respondents).

¶ Poverty status was assessed in National Survey on Drug Use and Health since 2003. Poverty status indicates a person’s family income relative to Federal poverty thresholds. https://www.census.gov/data/tables/time-series/demo/income-poverty/historical-poverty-thresholds.html.

** The Kessler Serious Psychological Distress is a series of six questions that asks about feelings of sadness, nervousness, restlessness or fidgetiness, worthlessness, hopelessness, and feeling like everything is an effort during the past 30 days. Responses were scored on a Likert Scale ranging from “None of the time” (score = 0) to “All of the time” (score = 4). Responses were summed over the six questions; scores could range from 0–24. Respondents with a score ≥13 were coded as having serious psychological distress, and respondents with a score <13 were coded as not having serious psychological distress. https://jamanetwork.com/journals/jamapsychiatry/fullarticle/207204.

†† Respondents were classified as being insured if they had private insurance, Medicare, Medicaid/HIPCOV, Champus, ChampVA, VA, Military, or other health insurance. Among veterans, weighted proportions of those insured and uninsured were 94.3% and 5.7%, respectively.

§§ Veteran and nonveteran populations differed in distributions of sex (males: 93.1% versus 43.2%, veterans and nonveterans, respectively) and age (persons aged ≥50 years: 76.2% versus 40.3%, veterans and nonveterans, respectively).

¶¶
https://www.publichealth.va.gov/smoking/quit/index.asp .

***
https://www.cdc.gov/tobacco/campaign/tips/groups/military.html .

†††
https://www.gpo.gov/fdsys/pkg/STATUTE-106/pdf/STATUTE-106-Pg4943.pdf .

§§§ https://www.publichealth.va.gov/smoking/quit/index.asp.

¶¶¶ http://www.med.navy.mil/sites/nmcphc/Documents/health-promotion-wellness/tobacco-free-living/INCOMING-CARTER-Tobacco-Policy-Memo.pdf.
